# Pharmacogenomics for Prediction of Cardiovascular Toxicity: Landscape of Emerging Data in Breast Cancer Therapies

**DOI:** 10.3390/cancers14194665

**Published:** 2022-09-25

**Authors:** Renske Altena, Svetlana Bajalica-Lagercrantz, Andri Papakonstantinou

**Affiliations:** 1 Department of Oncology-Pathology, Karolinska Institutet, 17 177 Stockholm, Sweden; 2 Department of Breast cancer, Endocrine tumors and Sarcoma, Theme Cancer, Karolinska University Hospital, 17 176 Stockholm, Sweden; 3 Department of Pathology and Cancer Diagnostics, Karolinska University Hospital, 17 176 Stockholm, Sweden; 4 Breast Cancer Group, Vall D’Hebron Institute of Oncology (VHIO), 08035 Barcelona, Spain

**Keywords:** breast cancer, cardiotoxicity, pharmacogenomics, anti-HER2, chemotherapy, immune checkpoint inhibitors, hormonal therapy, single-nucleotide polymorphisms (SNPs), polygenic risk scores

## Abstract

**Simple Summary:**

With the increasing number of breast cancer survivors and with the progress in survival of patients with metastatic breast cancer, consideration of survivorship issues becomes vital. Cardiotoxicity has been an important aspect in the management of early or metastatic breast cancer, not least due to the wide use of anthracyclines and, in HER2-positive breast cancer, anti-HER2 agents. Current baseline assessment includes clinical, such as history of cardiovascular comorbidities and lifestyle factors, and biochemical markers. Further biomarkers for tailored risk assessment and management remain an unmet need. Pharmacogenomics, an emerging field investigating how individual genetic variations, alone or in combination with polygenic risk scores, can impact drug metabolism and efficacy, and could be a complement. We hereby present a comprehensive review of the literature and the current landscape on the role of pharmacogenomics in cardiotoxicity prediction and its potential to become an additional biomarker in personalized risk assessment algorithms.

**Abstract:**

Pharmacogenomics is an emerging field in oncology, one that could provide valuable input on identifying patients with inherent risk of toxicity, thus allowing for treatment tailoring and personalization on the basis of the clinical and genetic characteristics of a patient. Cardiotoxicity is a well-known side effect of anthracyclines and anti-HER2 agents, although at a much lower incidence for the latter. Data on single-nucleotide polymorphisms related to cardiotoxicity are emerging but are still scarce, mostly being of retrospective character and heterogeneous. A literature review was performed, aiming to describe current knowledge in pharmacogenomics and prediction of cardiotoxicity related to breast cancer systemic therapies and radiotherapies. Most available data regard genes encoding various enzymes related to anthracycline metabolism and HER2 polymorphisms. The available data are presented, together with the challenges and open questions in the field.

## 1. Introduction

About one-third of cancer cases in women are attributed to breast cancer, although incidence can vary between geographic regions [1]. Breast cancer incidence is projected to rise in the coming decades because of ageing and the increase in the global population [2], and simultaneously, the number of breast cancer survivors is expected to rise as a result of the advances in understanding the biology and management of breast cancer. Multimodal care is today the standard in Western countries, including a plethora of combinations of surgery, conventional chemotherapy, targeted therapies (including antihormonal therapy and anti-human epidermal growth factor receptor 2 (HER2)-agents), and radiotherapy.

The development of cardiotoxicity, both acute and chronic, represents a substantial source of short- and long-term morbidity and may drastically impact quality of life in breast cancer survivors. Cardiotoxicity is a broad term that encompasses either direct cardiovascular adverse effects or progression of established cardiovascular disease [3]. A reduction of left ventricle ejection fraction (LVEF) by 10%–15% points and/or to a value below the lower normal limit by echocardiography or multigated acquisition scan (MUGA) is often used to diagnose cancer-therapy-related cardiac dysfunction [4,5,6].

Efforts to ameliorate cancer-therapy-related cardiac dysfunction is vital to decrease the risk for acute morbidity, chronic health conditions, and decreased quality of life in cancer survivors. Several strategies have been explored, for example, by means of early risk stratification or medical interventions with cardioprotective drugs.

Early risk stratification is key to identifying the optimal candidates for intensified screening programs, treatment modifications, or initiation of cardio-protective strategies. Traditional risk factors associated with risk for cardiotoxicity include increasing age; smoking; obesity; lack of physical activity; comorbidities such as diabetes mellitus; hypertension; hyperlipidemia and pre-existing or history of cardiac conditions; type of cancer therapy; chest irradiation; and, in the case of anthracyclines, cumulative dose and concomitant administration with trastuzumab [3,6]. The European Society for Medical Oncology (ESMO) recommends baseline screening with electrocardiogram and evaluation of LVEF for all patients planed for cancer therapy, especially if the planned treatment is correlated to increased risk for cardiotoxicity [6]. The ground for the use of biomarkers such as troponin and brain natriuretic peptide (BNP) or the N-terminal prohormone of BNP (NT-proBNP) is not equally robust and they are mostly recommended for patients with high baseline risk for cardiovascular side effects [6,7].

Existing evidence for cardiotoxicity prevention with angiotensin-converting enzyme inhibitors, angiotensin receptor blockers, or beta blockers have not yet provided significant clinical benefit, and interpretation is hampered by heterogeneity of the study populations and their baseline cardiotoxicity risk level, as well as of the definition of study endpoints. This inconsistency of the results in various randomized trials, often stratified by common risk factors, highlights the need for additional, objective, personalized, and risk-predictive biomarkers [8]. Ideally, a patient with increased risk for toxicity should be identified prior the initiation of a potentially cardiotoxic cancer treatment, allowing for modification of the planned treatment; initiation of preventive strategies; and, if necessary, establishment of a tailored monitoring schedule.

In this context, pharmacogenomics, an emerging field in clinical practice as well as in research, could be of great value. Pharmacogenomics examines how various genetic variants impact pharmacokinetics, i.e., how different genetic variants may influence the expression and function of the drug-metabolizing enzymes, as well as pharmacodynamics, i.e., how variants can affect expression of the drug targets and thereof the effect of the drug in the body. In this way, pharmacogenomics can provide insight on insufficient drug efficacy, variations in drug-related side effects, and drug-to-drug interactions. For example, allelic variations leading to high enzymatic cytochrome P450 family 2 (CYP2C19) and low CYP2D6 activity have been related to poor bioactivation of tamoxifen and subsequently worse breast cancer outcomes [9,10]. Additionally, in breast cancer patients treated with docetaxel, those with genetic variants leading to poor drug metabolism have been shown to have increased incidence of grade 3/4 toxicity (71%), compared to 45% among intermediate metabolizers and similarly to extensive or rapid metabolizers [11]. Hence, there is a strong rationale that allelic variants, single-nucleotide polymorphisms (SNPs), or substitution variants in processes involved in the function or metabolization of a drug could predispose to cardiotoxicity and could therefore serve as biomarkers for the prediction of treatment-related cardiotoxicity.

The utility of pharmacogenomics in the field of cardio-oncology is unclear thus far and the data are scarce. To this aim, we conducted a comprehensive literature review and present the available data on the predictive role of genetic variants for the development of acute and chronic cardiotoxicity in breast cancer survivors.

## 2. Chemotherapy

Anthracyclines and taxanes constitute the most commonly used regimens in early and metastatic breast cancer. Although the position of anthracyclines in neoadjuvant management of HER2-positive breast cancer is currently debated [12], their position in breast cancer care remains important. Anthracycline-related cardiotoxicity is described as a dose-dependent and irreversible cardiomyopathy, progressively leading to heart failure, and it was described already in 1967 [13]. Still, the exact pathogenic mechanism is not entirely known. Generation of reactive oxygen species (ROSs) and changes in iron metabolism were initially suggested as potential pathways, but in recent years, inhibition of topoisomerase 2β and subsequent mitochondrial dysfunction and cell death has been the most popular hypothesis [14,15].

In preclinical models using patient-derived cardiomyocytes, a missense variant S427L (rs2229774) of the retinoid acid receptor gamma (*RARG*) gene increased susceptibility to doxorubicin and subsequent cardiotoxicity [16].

Cytosolic carbonyl reductases (CBR) are enzymes involved in the metabolism of doxorubicin, and it has been demonstrated that different genotypes can significantly impact cardiotoxicity incidence [17]. In 92 breast cancer patients genotyped for the *CBR3* gene, those with the *CBR3* V244M variant had a significant reduction of LVEF six months after the initiation of doxorubicin, although the numerical reduction of LVEF was small [17]. Risk for cardiotoxicity, defined as asymptomatic LVEF decline <55%, did not correlate to any *CBR3* V244M genotype [17]. The same variant was also identified as a risk variant in a retrospective series of n = 166 patients with breast cancer treated with doxorubicin [18]. They also demonstrated that the variant rs1045642 of the ATP binding cassette subfamily B member 1 (*ABCB1*) gene, encoding for the P-glycoprotein that is involved in transportation of the drug outside the cell, was found to be related to lower risk for cardiotoxicity (odds ratio (OR) 0.48; 95% confidence interval (CI): 0.23–1.00; *p* = 0.049) [18]. Another variant, namely, *ABCC1* rs246221, was also identified as being correlated with cardiotoxicity in a retrospective study of 877 women [19]. In fact, LVEF decline correlated to heterozygous carriers (OR 1.59; 95% CI 1.1–2.3) [19].

In a study of 427 Asian women, the uridine glucuronosyltransferase 2B7 (*UGT2B7)* codon 161 SNP (C > T) was correlated with lower incidence of cardiotoxicity, defined as LVEF reduction ≥10% from baseline to <53%, heart failure, acute coronary artery syndrome, or fatal arrhythmia, among patients that received anthracycline and taxane adjuvant chemotherapy [20]. The TT homozygous alleles had the lowest incidence of cardiotoxicity (1.1%), followed by the heterozygous (3.1%), and finally the CC homozygous alleles, which were associated with the highest incidence (7.8%) [20]. In a multivariate analysis, the presence of a T-allele was found to be predictive of cardiotoxicity development, even when corrected for other known risk factors.

A meta-analysis, based on eight studies, investigated anthracycline-related cardiotoxicity (n = 84 genes related to n = 147 SNPs) and identified polymorphisms in three additional genes: (i) the ATP binding cassette subfamily C member 2 (*ABCC2*) gene, which encodes for the multidrug resistance protein 2 and is also involved in drug transport extracellularly (SNP rs8187710 OR 2.2; 95% CI 1.36–3.54); (ii) the cytochrome b-245 alpha chain (*CYBA*) gene that encodes a subunit of the NADPH oxidase enzyme complex (SNP rs4673 OR 1.55; 95% CI 1.05–2.30); and (iii) the Rac family small GTPase 2 (*RAC2*) gene that encodes for proteins that are involved in the generation of reactive oxygen species, among others (SNP rs13058338 OR 1.79; 95% CI 1.27–2.52) [21].

Another recent systematic review and meta-analysis on pharmacogenomic predictors for chemotherapy-induced cardiotoxicity included 41 studies, and all but three reported on anthracycline-containing regimens [22]. Among the 17 trials that were included in a meta-analysis, 14 SNPs were detected, of which 6 were found to be significantly associated with increased cardiotoxicity. Even here, some findings were consistent, demonstrating increased cardiotoxicity incidence in *CYBA* rs4673 polymorphism in *ABCC2* rs8187710 and in *RAC2* rs13058338. Additionally, the polymorphism rs776746 of the *CYP3A5* gene and the *ABCC1* rs45511401 polymorphism were also identified to be involved. SNPs reported to significantly affect risk for cardiotoxicity are summarized in Table 1.

## 3. Targeted Therapies

### 3.1. Anti-HER2

#### Trastuzumab

Anti-HER2-therapy-induced cardiotoxicity is considered dose independent and largely reversible, without structural cardiomyocyte damage, and usually improves after treatment discontinuation and adequate cardiological care [3]. Despite being considered reversible, the risk of anti-HER2-related cardiotoxicity increases with the administration of anthracyclines concomitantly or in sequence within a short interval [23]. Treatment with trastuzumab has been described as disrupting the recovery of the cardiomyocytes and can lead to accentuated remodeling of the myocardium and fibrosis. Both in the adjuvant and metastatic setting, pertuzumab added to trastuzumab did not have a negative impact on cardiac safety [24,25], and trastuzumab emtansine (T-DM1) has also shown low cardiotoxicity potential [26,27].

A meta-analysis of 35 published trials by Leong et al. [28] identified 74 SNPs suggested to be related to cancer-therapy-induced hypertension, decrease in LVEF, and venous thromboembolism, and one in particular (*HER2* codon 655 allele rs1136201) to augmenting the risk of trastuzumab-induced cardiotoxicity, as also demonstrated by a previous meta-analysis by the same group [21,28].

In a prospective observational study, the same *HER2* codon 655 A > G polymorphism (or rs1136201) was also related to increased cardiotoxicity in an Asian population (univariate analysis OR 3.1, *p* = 0.008) [29]. In total, 91 patients were included and were followed during adjuvant therapy consisting of an anthracycline regimen first and subsequent initiation of trastuzumab. Twenty-six patients developed cardiotoxicity defined as LVEF decline of 10% from baseline and below 53% or on the basis of clinical criteria, i.e., diagnosis of heart failure, acute coronary artery syndrome, or fatal arrhythmia [29]. Carriers of the *HER2* codon 655 A > G were eight times more likely to develop cardiotoxicity according to a multivariate analysis (*p* = 0.007).

On the contrary, a series with 140 Caucasian patients reporting findings of *HER2* polymorphisms did not find a significant correlation of the codon 655 polymorphism and cardiotoxicity. The analysis was based on 29 patients with cardiotoxicity during adjuvant trastuzumab, defined as symptomatic heart failure or LVEF decline of 15% or of 10% if LVEF was less than 55%, as well as 111 controls [30]. An age-, ethnicity-, and hypertension-status-adjusted multivariable analysis demonstrated a correlation between the proline 1170 polymorphism of *HER2* and risk for cardiotoxicity (adjusted OR = 2.60; 95% CI = 1.02 to 6.62, *p* = 0.046) [30]. However, no data on chemotherapy, hormonal therapy, or radiotherapy administration are provided, impeding correlation with other known confounding factors.

Similar findings were reported in a cohort of 177 European patients, 78 of which had cardiotoxicity defined according to LVEF decline as above [31]. Although mean LVEF among patients with *HER2* codon 655 GG homologous allele was lower during adjuvant trastuzumab, this observation was not statistically significant. Of note, the 99 control patients were selected by chance, resulting in baseline imbalances between the groups. Twice as many patients with LVEF decline had left-sided breast cancer (60% vs. 36%, *p* = 0.002), which per se increases cardiotoxicity risk [31]. Additionally, the incidence of family history of heart disease (40 vs. 20%, *p* = 0.004) and lymph node burden (11% vs. 1%) were significantly higher in this group. Although not statistically significant, more patients in the control group had received non-anthracycline-containing regimens (13% vs. 8%).

A meta-analysis of four series with a total of 344 patients that received trastuzumab reported more than a five times higher risk of cardiotoxicity (varying definitions per study) in patients with the *HER2* codon 655 AG polymorphism compared to the AA homologous one (OR 5.35; 95% CI 2.55–11.73, *p* < 0.0001) [32]. The AG polymorphism was significantly related to increased cardiotoxicity incidence in three of the four included cohorts [32,33,34,35]. The series published by Beauclair et al. did not report odds ratios, and interestingly, it was the only cohort with patients receiving trastuzumab in the metastatic setting [33].

Of note, the above-mentioned HER2 codon 655 polymorphism (rs1136201) has also been investigated as a breast-cancer-susceptible SNP, but this hypothesis has not been confirmed. Two small case–control studies in Austrian and Brazilian populations did not demonstrate a significant increase in breast cancer risk in patients carrying the SNP [36,37]. Moreover, a systematic review and meta-analysis also reported lack of such a relationship [38], although studies in controlled conditions and large populations would probably be required to safely exclude such a relationship.

Recently, Peddi et al. reported genotyping results from probably the largest dataset thus far, with 666 of the 3222 patients with early HER2-positive breast cancer included in the Breast Cancer International Research Group (BCIRG)-006 trial [39]. Of them, 224 patients received anthracycline and trastuzumab therapy in sequence, 226 did not receive anthracyclines, and 216 did not receive trastuzumab. Cardiac dysfunction was defined as LVEF decline of at least 10%, but also a broader definition of symptomatic heart failure was also used in a secondary analysis. No significant difference was seen among the investigated polymorphisms, rs1136201 (*HER2* codon 655) and rs7853758 (*SLC28A3* gene), although the AG heterologous allele demonstrated a small but significantly increased probability of cardiotoxicity when the group treated with anthracyclines and trastuzumab in sequence was analyzed separately [39].

The antioxidative-enzyme-encoding genes paraoxonase 1 (*PON1*), glutathione-S-transferases (*GSTs*), and catalase (*CAT*) stood out in a retrospective series of 101 patients with HER2-positive breast cancer that received adjuvant trastuzumab [40]. The vast majority (98%) of the patients had received anthracycline-containing regimens prior to trastuzumab administration, and 84% of them also underwent adjuvant radiotherapy, half of which was towards the left chest. In total, 36 patients had NT-proBNP equal to or higher than 125 ng/L [40], and two alleles of the *PON1*-gene were identified with opposing findings. Patients with at least *PON1* rs662 polymorphism were characterized by significantly increased (≥125 ng/L) NT-proBNP at completion of the adjuvant trastuzumab (age-adjusted OR 5.41; 95% CI 2.12–13.78; *p* < 0.001), whereas those with *PON1* rs854560 had significantly lower median NT-proBNP level (age-adjusted OR = 0.35; 95% CI 0.15–0.83; *p* = 0.017) [40]. In this study, LVEF levels did not correlate to any particular SNPs, but it is worth noting that there were only nine patients with LVEF lower than 55%, impeding any conclusions. Finally, carriers of the allele *CAT* rs1001179 were more likely to have symptoms comprising New York Heart Association (NYHA) class 2 (adjusted OR = 4.14, 95% CI 1.22–14.09).

### 3.2. CDK-4/6 Inhibitors

The cycline-D kinase 4/6 inhibitors palbociclib, abemaciclib, and ribociclib have revolutionized the management of metastatic estrogen receptor (ER)-positive breast cancer and enabled chemotherapy-free early line treatment regimens for patients without significant organ impairments due to their metastases, with comparable disease-related outcomes [41,42].

In the MONALEESA-2 trial comparing ribociclib plus letrozole to letrozole alone as first-line therapy, ribociclib was linked to QTc prolongation in 2.7% of the patients and none in the placebo-treated group [43]. In general, 3.3% of the ribociclib-treated patients experienced QTc interval prolongation of more than 480 ms. This finding does not seem to be a class action since the rest of the CDK4/6 inhibitors have thus far not demonstrated similar properties. Moreover, CDK4/6 inhibitors have not demonstrated significant increase in other types of cardiovascular toxicity such as coronary artery disease, thrombo-embolic events, or cardiomyopathy [44,45].

To the best of our knowledge, the possible predictive role of specific genotypes for ribociclib-associated QTc-prolongation has not been investigated. However, genetic variations known to be associated with drug-induced QTc-prolongation could probably be used to identify persons of increased risk, as described for anti-psychotic and thiazide-induced QTc-prolongation [46,47]. Candidate genes in this regard could be those encoding drug-metabolizing cytochrome P450 enzymes, drug transporters, or genes associated with QT interval duration and those correlated with congenital long QT syndromes [48]. For example, CYP3A is a cytochrome P450 enzyme essential for the hepatic metabolism of ribociclib, as well as palbociclib, and polymorphisms of the *CYP3A* gene could directly affect risk of toxicity and thereof QT_c_ prolongation [49,50]. Future studies will hopefully shed light on this issue and aid in identifying patients at specifically high or low risk for QTc prolongation related to ribociclib and the risk for severe ventricular arrythmias.

### 3.3. Phosphatidylinositol 4,5-Bisphosphate 3-Kinase Catalytic Subunit Alpha Isoform (PIK3CA) Inhibitors

The PIK3CA inhibitor alpelisib was approved by the FDA in 2019 for administration in the metastatic setting, on the basis of the findings from the phase 3 Solar-1 trial [51,52,53]. Preclinical studies have suggested that PIK3CA inhibitors may be related to arrythmias, in contrast to drugs that inhibit other PI3K isoforms, through the induction of late sodium current [54,55]. Notably, the rate of cardiovascular events in the Solar-1 trial was very low: any grade cardiac arrest (one in the intervention group), cardiac failure (two in the control group), myocardial infarction (one in the control group), and sinus tachycardia (one in the control group) [51]. However, hyperglycemia is a frequently observed side effect, also known to increase the risk of cardiovascular morbidity in the long term. We were not able to identify studies that have investigated the relationship between the risk of treatment-related toxicity and specific genotypes of PIK3CA inhibitors.

### 3.4. Mammalian Target of Rapamycin (mTOR) Inhibitors

The results of the Breast Cancer Trials of Oral Everolimus-2 (BOLERO-2), comparing exemestane with and without the mTOR inhibitor everolimus, led to the approval of the combination therapy for the treatment of metastatic ER-positive breast cancer [56]. Everolimus has not been described as directly inflicting cardiac toxicity but is associated with increased risk of hyperglycemia, hyperlipidemia, and hypertension—known risk factors for cardiovascular disease [57].

In a study of 90 patients with metastatic breast cancer treated with everolimus and the aromatase inhibitor exemestane, SNPs (n = 12) in genes involved in everolimus pharmacokinetics and pharmacodynamics were genotyped and correlated with drug levels in the plasma and toxicity [58]. In this cohort, the CYP3A4*22 allele influenced plasma concentration of everolimus, and other SNPs were associated with treatment-related toxicities. However, no relationship between these genotypes and occurrence of cardiovascular events were reported in this study, and other data on cardiotoxicity are also lacking. CYP3A4*22 polymorphisms could, however, be candidates for future pharmacogenomic investigations on mTOR inhibitors and cardiotoxicity risk prediction.

### 3.5. Immune Checkpoint Inhibitors

Myocarditis in patients treated with immune checkpoint inhibitors is rare, occurring in about one percent of the patients and having an early onset, but its high mortality rates (50%) call for attention [59,60,61]. Importantly, no cases of myocarditis were reported in patients with advanced triple-negative breast cancer treated with atezolizumab or pembrolizumab in the Impassion130 and Keynote-522 trials, respectively, nor with pembrolizumab in the neoadjuvant setting in Keynote-355 [62,63,64]. Cytotoxic-T-lymphocyte-associated antigen-4 (*CTLA-4*) polymorphisms rs4553808, rs11571317, and rs231775 have been associated with response to therapy with ipilimumab for metastatic melanoma, and the *CTLA-4* rs4553808 has also been correlated with higher incidence of endocrine side effects [65,66]. It is not possible to extrapolate whether the same polymorphisms could be predictive of cardiotoxicity, and given the rarity of the adverse event, it will probably be burdensome to map it, albeit important. Hence, we would like to suggest investigating *CTLA-4* polymorphisms as potential predictors of immune-checkpoint-inhibitor-related myocarditis and cardiovascular toxicity.

Moreover, taking into consideration the mechanism of action of the checkpoint inhibitors, SNPs in genes related to the susceptibility loci of the major histocompatibility complex (MHC) could be potential candidates in the context. Polymorphisms shown to be related to autoimmune diseases have been suggested as potential predictive biomarkers of immune-related cardiotoxicity and other toxicities, but data supporting this hypothesis are currently lacking [67].

## 4. Endocrine Therapy

In general, cardiovascular effects of antihormonal drugs such as aromatase inhibitors (AIs) and selective estrogen receptor degraders (SERDs) seem to be relatively modest. In a large meta-analysis that included 19 RCTs with N = 62,345 patients treated with antihormonal therapies in the adjuvant setting after breast cancer surgery, AIs had a 19% (relative risk (RR) 1.19, 95% CI 1.07–1.34) increased risk of cardiovascular events compared with tamoxifen [68]. However, in this analysis, AIs were not associated with an increased risk for cardiovascular events compared with placebo in the extended-adjuvant setting (RR 1.01, 95% CI 0.85–1.20) [68]. Interestingly, in the adjuvant setting, tamoxifen was associated with a 33% (RR 0.67, 95% CI 0.45–0.98) decreased risk for cardiovascular events compared with placebo or no treatment [68]. On the basis of these data, it has been concluded that tamoxifen probably has cardioprotective effects.

SNPs in, for example, the drug metabolic enzyme cytochrome P450 19A1 (*CYP19A1*) and the estrogen receptor 1 (*ERS1*) gene have been related to the risk for AI-related side effects, but mainly in relation to the development of arthralgias, menopausal symptoms, and osteoporosis [69,70,71]. No studies identified as having explored the risks for cardiovascular events according to genetic polymorphisms have been identified.

However, we did identify one study that investigated the relationship between specific genotypes and the risk of the development of venous thrombosis related to treatment with the tamoxifen. In a study of 220 patients treated with tamoxifen, women with the XbaI (rs9340799) genotype and *ESR1* Xbal/PvuII diplotype (rs9340799 and rs2234693) had an increased risk of a hazard ratio of 3.47 for the developing tamoxifen-associated thromboembolic events TTE (HR 3.47, 95% CI 0.97–12.44), results that persisted even after correction for other risk factors for TTE [72].

## 5. Radiotherapy

Left-sided chest radiotherapy is a known risk factor for short- or long-term cardiotoxicity. Radiotherapy can impact all layers of the heart (myocardium, pericardium, and endothelium) and can also lead to coronary artery stenosis [73]. In 57 breast cancer patients that underwent short partial breast irradiation (total dose 18–21Gy), a variant of the glutathione S-transferase pi 1 (*GSTP1)* gene was related to increased probability of grade 2 or above fibrosis or fat necrosis [74]. Similar analyses for cardiotoxicity and coronary artery stenosis are not available, but they should be considered.

## 6. Polygenic Risk Scores

The above-mentioned studies mainly focused on a few genetic markers associated with different degrees of cardiotoxicity. Genetic risk, however, most commonly constitutes of a combination of intermediate- and low-risk variants rather than a few high-risk genetic alterations [75], and risk prediction scores based on genome-wide association studies have been suggested to provide valuable information at the individual level [76]. A polygenic risk score including 1,745,180 variants was associated with higher risk of coronary artery disease in women with breast cancer, without baseline risk factors, even after adjustment for sociodemographic and medical confounders, including type of oncological treatment (HR 1.33; 95% CI 1.20–1.47) [77]. Among the 12,413 women included in this analysis, 38% had received adjuvant chemotherapy and 71% had received adjuvant radiotherapy. Moreover, adding a polygenic risk score to validated clinical risk scores for coronary artery disease in a large population improved the accuracy of risk prediction [78]. Although no information is provided on the individual risk of chemotherapy- or radiotherapy-related coronary artery disease or other types of cancer-therapy-induced cardiovascular toxicity, these two examples provide evidence of the usefulness of polygenic risk scores in the tailoring of the baseline risk assessment of the individual patient.

In addition to assessing the combined genetic risk from the PRS, co-medication and the impact of the interaction between different drugs and genes (drug–drug–gene interaction) has to be considered and is by far, less understood [79]. Interactions between co-administered drugs, interactions between genes, and drug–drug–gene interactions would need to be assessed in an algorithm for an improved cardiotoxicity risk prediction.

## 7. Discussion

The development of early and late cardiotoxicity is a potentially serious complication of breast cancer therapies that can lead to morbidity and can profoundly affect quality of life for cancer patients and survivors. Following improvement of breast cancer survival, there is an increasing number of breast cancer survivors, and thus diminishing long-term cardiotoxicity is essential. Estimation of the risk of development of cardiac toxicities from different cancer therapies should preferably be conducted prior to the initiation of therapy, and in this regard, pharmacogenomics provides a promising strategy that enables an individualized approach in establishing the risk–benefit balance for patients. This approach is currently advocated for in example fluoropyrimidine treatments, where SNPs of the gene of the drug metabolizing enzyme dihydropyrimidine dehydrogenase (DPD) are routinely assessed in many countries and, accordingly, dose modifications are considered [80].

We reviewed the literature for studies investigating the role of pharmacogenomics in identifying the risk level of cancer-therapy-related cardiotoxicity in breast cancer patients. Most studies involved patients treated with anthracyclines and/or HER2-targeted therapies, and modest correlations were found for specific variants in genes involved in drug metabolism, transport, and reactive oxygen species (ROS). Variants in such genes can modify the risk for development of cardiotoxicity through different mechanisms, for example, by altering the pharmacokinetics and pharmacodynamics of drugs that in turn lead to higher or prolonged drug plasma concentrations. Differences in the formation of ROS, which are thought to be one of the pathogenic factors in the development of anthracycline-induced cardiotoxicity, may lead to higher levels of ROS and thereby modify the risk for developing the specific toxicity.

Moreover, the polymorphism of the HER2 655 codon from A to G results in a valine (Val) amino acid instead of isoleucine (Ile), leading to a decreased tyrosine activity and thereby increased cell sensitivity to trastuzumab [81]. This could in turn lead also to increased sensitivity to adverse effects of trastuzumab on cardiomyocytes. Various studies have investigated correlations between HER2 655 polymorphisms and the risk of cardiotoxicity in patients treated with trastuzumab, but the findings of small retrospective series were not confirmed in a larger prospective cohort [39].

On the basis of the available data, no practical recommendations can yet be made to implement the use of pharmacogenomic strategies to ameliorate the risk for cardiac toxicity of very frequently used systemic drugs such as anthracyclines and trastuzumab in breast cancer management. Apparent issues related to the lack of conclusive findings may relate, amongst others, to the lack of a uniform definition of cardiotoxicity between different studies, as well as to heterogeneity in given systemic therapies. In addition, other candidate SNPs might prove to be more relevant, e.g., variants related to the development of other cardiac diseases such as dilated cardiomyopathy [82].

**Figure 1:**
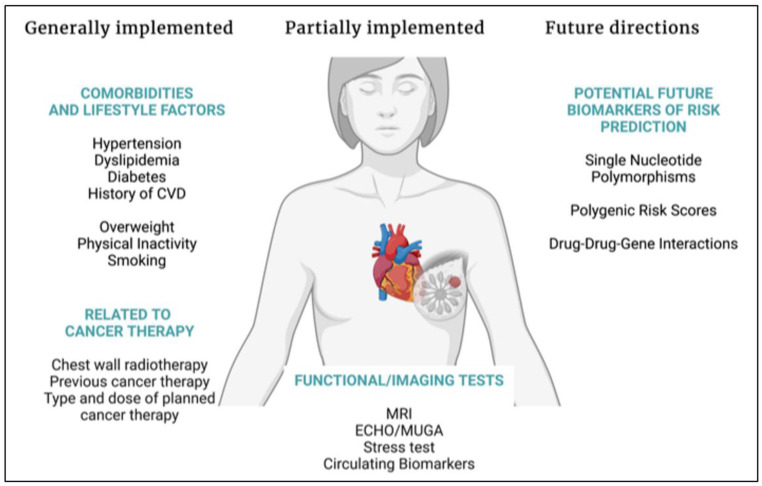
Summary of factors to be assessed concordantly with oncological treatment planning. Abbreviations: CVD: cardiovascular disease, ECHO: echocardiogram, MRI: magnetic resonance imaging, MUGA: multigated acquisition scan. Created with BioRender.com.

Data on relations between specific genetic variations and the development of cardiotoxicity from new-generation targeted therapies available in the management of breast cancer are still scarce. In the long term, pharmacogenomics could become a compliment to known and established stratification factors, not only for cardiotoxicity risk assessment but also for other types of severe and dose-limiting toxicities. A possible integrative model that could consist of a baseline assessment including established clinical risk factors for cardiovascular disease, testing for selected nucleotide polymorphisms, or individualizing treatment strategies on the basis of polygenic risk score calculations, as well as taking into consideration the expected benefits and risks of the oncological treatment indicated for the individual patient is presented in Figure 1.

This model could assist in the identification of the patients at most risk and inform us of the stratification for future studies investigating the utility of preventive strategies, as for example with beta-blocking agents or angiotensin receptor antagonists. This is an area that warrants further investigation since the exact impact of primary prevention for the prevention of cancer-treatment-related cardiotoxicity is still unclear [8].

Combining data from the available studies is cumbersome and should be performed with caution. Limitations in the comparison of the studies included in this review have been the heterogeneity in the cardiotoxicity definition, the methodology used to identify candidate genes and SNPs (wide versus directed analysis), the patient characteristics in each cohort and varying baseline risk for cardiotoxicity, and finally the heterogeneity in the assessed timepoints and length of follow-up. The advances in breast cancer management, as for example the shift to administrating anthracyclines and anti-HER2 agents in sequence and not concomitantly, as well as the increasing popularity of de-escalation efforts with anthracycline-free regimens in patients, will lower the risk for toxicity, and advances in radiotherapy techniques are expected to further decrease cardiotoxicity incidence. Thereof, small retrospective cohorts probably lack the power to identify genetic polymorphisms and undermine their significance. However, the use of pharmacogenomic parameters to predict and thereby prevent treatment-associated cardiotoxicity is tempting. There is an indisputably unmet need for prospective studies with well-defined patient populations, including a baseline cardiovascular phenotype, and outcome measures.

Suggestions for future studies include harmonization, including the definition of therapy-related cardiac dysfunction and the length of follow-up, and expansion of outcomes, including subclinical markers for cardiotoxicity. Additional candidate SNPs might be considered, e.g., genes known to predispose for other cardiac conditions, as well as enhanced/deepened explorations that take drug–drug–gene interactions into account [79]. The latter relates to potentially synergistically or antagonistically acting interactions that may further modify risks for treatment-related toxicities.

## 8. Conclusions

Moderate correlations with a number of candidate SNPs were found in relation to the development of cardiotoxicity caused by anthracyclines and/or HER2-targeted therapies, whereas data for other therapies is currently scarce, at best.

To facilitate clinical implementation of pharmacogenomics to predict cancer-treatment-related cardiotoxicity, future research efforts should aim to expand comparable approaches to other candidate SNPs as well as additional treatments such as checkpoint inhibitors and CDK4/6 inhibitors, and to include additional acute and chronic cardiotoxicity risk factors with the aim of creating a comprehensive baseline risk assessment. This will aid in further treatment tailoring and enhancing healthy cancer survivorship.

## Figures and Tables

**Table 1:** Study characteristics, endpoint definition, and important findings in correlating genetic polymorphisms and risk for cardiotoxicity.

Author, Year	Number of Patients	Gene	SNPs	Correlation to Cardiotoxicity Endpoint	Odds Ratio (OR) (95% CI)	Definition of Cardiotoxicity Endpoints
Drug: anthracyclines						
Yang, 2021	41 studies	*CYBA* *RAC2* *CYP3A5* *ABCC1* *ABCC2* *HER2* codon 655	rs4673 rs13058338 rs776746 rs45511401 rs8187710 rs1136201	⇑ ⇑ ⇑ ⇑ ⇑ ⇑	1.93 (1.13–3.30) 2.05 (1.11–3.7) 2.15 (1.00–4.62) 1.46 (1.05–2.01) 2.19 (1.38–3.48) 2.48 (1.53–4.02)	Depending on the study
Leong, 2017	28 studies	*ABCC2* *CYBA* *RAC2*	rs8187710 rs4673 rs13058338	⇑ ⇑ ⇑	2.2 (1.36–3.54) 1.55 (1.05–2.30) 1.79 (1.27–2.52)	Depending on the study
Vulsteke, 2015	877	*ABCC1*	rs246221	⇑	1.6 (1.1–2.3)	Asymptomatic decrease in LVEF >10% or cardiac failure grade ≥3
Li, 2019	427	*UGT2B7* codon 161	rs7668258	⇑	NR	LVEF decline ≥10% to <53%, heart failure, acute coronary artery syndrome, or fatal arrhythmia
Hertz, 2016	166	*CBR3* *ABCB1*	rs1056892 rs1045642	⇑ ⇓	NR	LVEF reduction to <55%
Lang, 2021	92	*CBR3*	rs1056892	LVEF reduction from baseline but no correlation to cardiotoxicity	2.55 (0.26–25.17) for GG vs. AA; 1.43 (0.15–13.26) for GA vs. AA	Asymptomatic LVEF reduction of ≥10% to levels < 55%
**Drug: trastuzumab**						
Leong, 2019	35 studies	*HER2* codon 655	rs1136201	⇔	2.43 (1.17–5.06)	LVEF decline ≥10% from baseline and <53% or heart failure, acute coronary artery syndrome, or fatal arrhythmia
Gómez Peña, 2015	4 studies	*HER2* codon 655	rs1136201	⇑	5.35 (2.55–11.73)	Depending on the study
Peddi, 2022	666	No polymorphisms correlated with cardiotoxicity were identified				LVEF decline ≥10% or symptomatic heart failure
Vazdar, 2021	177	*HER2* codon 655	rs1136201	⇔	NR	LVEF and diastolic dysfunction; cut-offs not clarified
Stanton, 2015	140	*HER2* *HER2* codon 655	Pro1170Ala rs1136201	⇑ ⇔	2.60 (1.02–6.62) NR	Symptomatic heart failure or LVEF decline ≥15% or LVEF decline ≥10% and <55% and discontinuation of trastuzumab (temporary or permanent)
Marinko, 2022	101	*PON1* *PON1* *GSTP1*	rs854560 rs662 rs1695	⇓ ⇑ ⇓	0.35 (0.15–0.83) 5.41 (2.12–13.78) 0.36 (0.15–0.88)	NT-proBNP increase to ≥125 ng/L
Tan, 2020	91	*HER2* codon 655	rs1136201	⇑	7.99 (95% CI NA; *p* = 0.007	LVEF decline ≥ 10% from baseline and <53%, or heart failure, acute coronary artery syndrome, or fatal arrhythmia

Abbreviations. ABCB1: ATP binding cassette subfamily B member 1, ABCC1: ATP binding cassette subfamily C member 1, ABCC2: ATP binding cassette subfamily C member 2, CBR3: cytosolic carbonyl reductases, CI: confidence interval, CYBA: cytochrome b-245 alpha chain, CYP3A5: cytochrome P450 family 3 subfamily A member 5, GSTP1: glutathione S-transferase pi 1, HER2: human epidermal growth factor receptor-2, LVEF: left ventricle ejection fraction, NA: not available, NT-proBNP: N-terminal prohormone of brain natriuretic peptide, NR: not reported, OR: odds ratio, PON1: paraoxonase 1, RAC2: Rac family small GTPase 2, SNP: single-nucleotide polymorphism, UGT2B7: uridine glucuronosyltransferase 2B7. ⇑: increases risk, ⇓: decreases risk, ⇔: no correlation.

## Data Availability

Not applicable.
